# Hydrochemical analysis and identification of open-pit mine water sources: a case study from the Dagushan iron mine in Northeast China

**DOI:** 10.1038/s41598-021-02609-0

**Published:** 2021-11-30

**Authors:** Qianling Liu, Zhongjian Zhang, Bin Zhang, Wenping Mu, Huijie Zhang, Yutao Li, Nengxiong Xu

**Affiliations:** 1 School of Engineering and Technology, China University of Geosciences (Beijing), Beijing, 100083 China; 2 Key Laboratory of Deep Geodrilling Technology, Ministry of Natural Resources, Beijing, 100083 China

**Keywords:** Environmental sciences, Hydrology, Geochemistry

## Abstract

The identification of open-pit mine water sources is of great significance in preventing water disasters. Combined with hydrochemistry and multivariate statistical analysis, this paper systematically analyzed the hydraulic connections between aquifers and the complex seepage water sources in the pit and roadway of Dagushan iron mine through qualitative analysis and quantitative calculation. According to the hydrochemical characteristics of the study area, the causes of seepage water at different positions in the mining area were reasonably explained. The results show that there is a possible hydraulic connection or similar source of water body between the bedrock fissure aquifer and the eluvium pore aquifer. The water seepage of 2# roadway mainly comes from bedrock fissure aquifer in the north of mining area. The reason for serious water seepage in the 3# roadway and the western side of the pit is that the fault connects the shallow alluvial pore aquifer and bedrock fissure aquifer. The source of water on the southern side pit comes from the river and groundwater on the southern side of the mine. The results presented here provide significant guidance for the management of mine water seepage problems.

## Introduction

Open-pit mining is the main method to obtain ore raw materials^1^. The excavation of pits and underground transportation roadways inevitably destroys the aquifer structure, affects the normal recharge circulation path of groundwater, and causes mine water seepage problems^2,3^. For mining activities, the long-term flow of groundwater in the open pit will affect mining operations and increase the cost of ore transportation, while reducing slope stability^4–7^. At present, the method of pumping and draining is mainly used for the treatment of seepage water in mining area. This method cannot effectively control the water disaster, but the long-term pumping will cause the gradual degradation of the ecological environment, including groundwater level decline^8^, water pollution^9,10^ and soil degradation^11–13^ around the mining area. Therefore, in order to ensure the safety, sustainability and economic return of underground mining, as well as to provide reasonable and effective prevention and control measures for water disasters, it is necessary to understand the hydraulic connections between different aquifers and to accurately identify the sources of open-pit mine water^14,15^.

Using conventional hydrochemical components in groundwater as natural tracers, relevant experts and scholars have proposed many mathematical methods for mine water source identification, such as fuzzy evaluation^16,17^, unascertained clustering average method^18^, grey correlation and stepwise discriminant analysis^19–21^, artificial neural network (ANN) and support vector machine (SVM)^22–24^. In these methods, the water seepage source is obtained by analyzing the calculation results of hydrochemical composition based on mathematical algorithm. There is a subjectivity, and the judgment process is complex. In addition, water sources can be accurately identified only when there is a large amount of hydrochemical data. Some scholars also proposed the methods that use groundwater level and water pressure monitoring^25^, water temperature measurement^26,27^ and organic matter content^28,29^ for mine water source identification. In these methods, the prerequisite for accurate water source identification is that a obvious difference between different aquifers exists, so they may not be applicable to water source identification of other mines.

The hydrochemical data are very easy to obtain in the mine because of low test fee and the high demand of hydrogeological exploration^27^. In the process of analysis, the sample data need not meet special conditions, and it is simple and effective to use multivariate statistical theory to determine the water source^30,31^. Regional hydrogeochemical controlling factors can be determined by studying hydrochemical parameters with graphical methods and ion ratio relationships^32^, and the hydraulic connections between different aquifers can be determined by source information for groundwater recharge and discharge^33–35^. Combining with different statistical analysis methods to classify hydrochemical data is helpful to understand the hydraulic relationship between groundwater in different aquifers and mine water, it is also a common method to determine mine water sources^34,36–39^. However, relevant studies ignore the further explain the results of multivariate statistical analysis in combination with hydrochemical characteristics, which is of great significance to improve the accuracy of identifying water sources in mining areas with complex seepage characteristics.

Based on the qualitative analysis and quantitative evaluation of the hydrochemical data in the mining area, the present paper studies the reasons for the formation of different groundwater characteristics and the recharge relationship, and reasonably explains the complex causes of water seepage in different locations. Firstly, the ion concentration characteristics and hydrochemical types of groundwater and seepage in the mining area are compared and analyzed. Then, the samples were grouped by hierarchical cluster analysis (HCA), and the hydraulic connections between groundwater in different aquifers and mine water were considered by analyzing the hydrochemical characteristics of each group. Finally, the discriminant model of mine water sources was established using Fisher’s discriminant analysis (FDA), and this, combined with the results of HCA and hydrochemical characteristics of groundwater in the study area, allowed the sources of seepage water in different locations of the mine to be determined. Mine water disaster control is a long-term problem accompanied by mining. The combination of hydrochemistry and multivariate statistics provides a strong basis for using "quantitative theory" to identify water seepage sources and has important guiding significance for the treatment of mine seepage water problems.

## Study area

### Physical geography and geology

The Dagushan iron mine is located in Anshan City, Liaoning Province, Northeast China. The general topographic relief of the study area is high in the southeast and low in the north. Qianshan Mountain with a maximum altitude of 673 m is found along the southeast side of the study area. The southern, eastern, and northern parts of the study area are composed of mountainous hills and alluvial plain. The western part of the study area is mainly composed of plains with an average altitude of approximately 80 m, besides low mountains and hills with an altitude of approximately 120 m near Zhangziwo. The study area is located in the temperate monsoon region with an average annual temperature of 8.8 °C. Rainfall mostly occurs between June and September, with a mean annual precipitation of 720.6 mm. The evaporation effect is strong, with an annual average evaporation of 1058.5 mm. Rivers distribute in the valleys in the southern and eastern parts of the study area; the flow of these rivers is relatively large in the historical period, and the widest riverbed is approximately 10 m wide. However, in recent years, the river flow has gradually decreased, and partially cut-off around the western and southern sides of the mine in the dry season.

The strata cropping out in the study area include the Archean Anshan Group, the Lower Proterozoic Liaohe Group, the Upper Proterozoic Qingbaikou system, the Upper Proterozoic Sinian system and the Cenozoic Quaternary overburden. The lithology in the study area is mainly granite, among which K-feldspar granite is widely distributed. The Anshan metamorphic rocks, including phyllite and quartz schist, are distributed widely along the northwest–southeast directions. The plain area is mainly covered by Quaternary sediments, comprising clay and sandy clay. The main iron orebody is stratiform and divided into the West orebody and the East orebody by diorite porphyry dike^40^.

**Figure 1:**
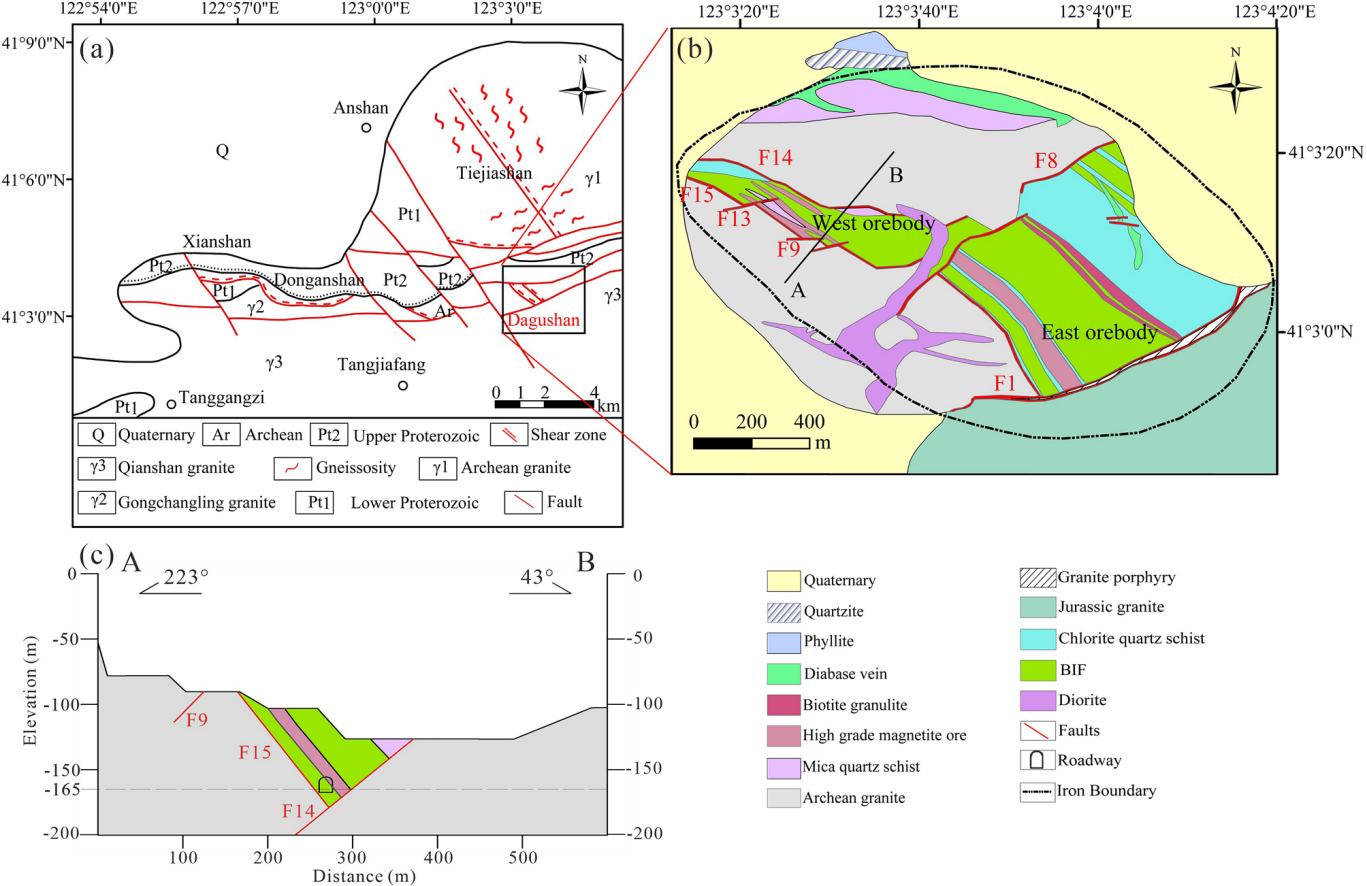
Geological setting of the study area. **(a)** Geological structure sketch map of the Anshan area (modified from^41–43^). **(b)** Geological structure of the mine area. **(c)** Geological section of the A–B.

Figure 1 showed geological setting of the study area. The study area is located in the Anshan-Benxi structural deformation belt, which belongs to the secondary fault at the east wall of the Tanlu fault zone in China and has experienced three stages of structural deformation: granite intrusion, ductile shear deformation and superimposed tectonic movement^41^.

Based on the open access data in the Geological cloud of China Geological Survey^42^ and Regional Geological Records of Liaoning Province^43^, and combined with the research from Guo (1994) ^41^, it is known that the Hanling fault composed of several parallel or intersecting faults has the greatest impact on the iron ore deposit (Fig. 1a). As shown in Fig. 1a, the faults are nearly parallel along the east–west direction, striking 280–310°. There are two main groups of faults associated with the orebody^44^. The first group of faults, F14 and F15 located in the western part of the mining area, represents the boundary between the western orebody and Archean granite (Fig. 1b). The dip directions of the faults are SW and NW-N, respectively, with dip angles of 50°–60°. In addition, this group of faults is cut by faults F9 and F13 with EW-strikes. The second group of faults, F1 and F8 located in the eastern part of the mining area, represents the boundary between Jurassic granite, diorite and the eastern orebody, striking SE and with dip angles of 40°–70°.

### Hydrogeological settings

**Figure 2:**
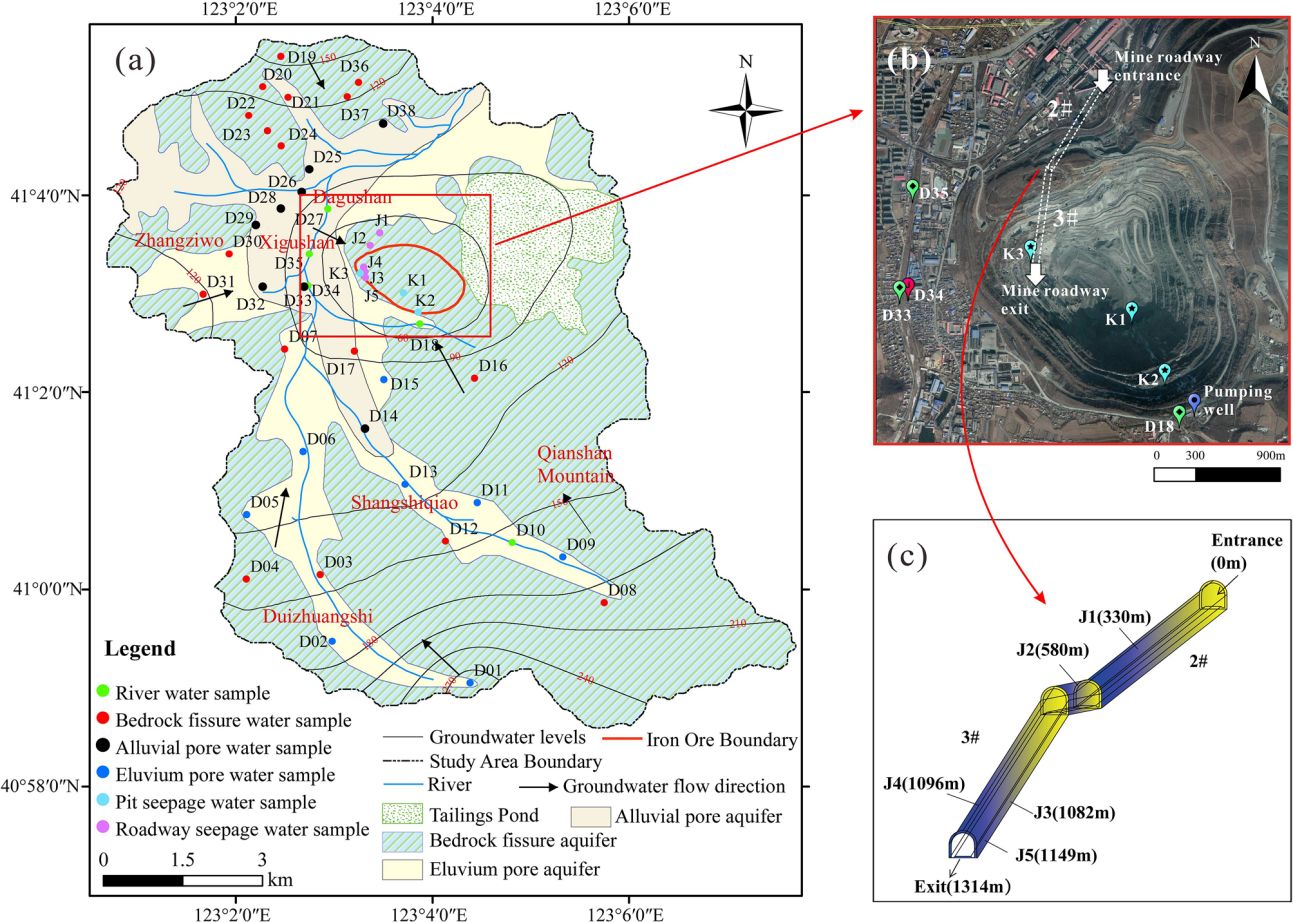
**(a)** Hydrogeological map of the study area and sampling points (the map was generated using ArcGIS 10.2, https://www.esri.com). **(b)** Satellite image illustration of water seepage points in the open-pit. **(c)** Schematic diagram of water seepage points in the roadway.

The aquifer in the study area can be divided into a Quaternary pore aquifer and a bedrock fissure aquifer according to stratum lithology (Fig. 2a). The Quaternary pore aquifer is subdivided into alluvial pore aquifer and eluvium pore aquifer. The alluvial pore aquifer is mainly composed of clay, sand and gravel, with thickness ranging from 17.9 to 29.0 m. The water level of the aquifer is 1.46–5.19 m. The specific well discharge ranges from 1.42 to 3.10 L/(s·m), which is considered as a high water yield property. The eluvium pore aquifer is distributed in valleys and foothills, with a thickness ranging from 1 to 10 m and composed of pebbles, gravels, and clay, with a weak water yield. The water level of the aquifer is 2.18–9.81 m. The bedrock fissure aquifer are mainly distributed in the hills and mountainous areas of the study area, primarily composed of Archean granite and K-feldspar granite. It contains weak weathering fractures and structural fractures. The main recharge source of groundwater is atmospheric rainfall and the groundwater flow has good connectivity. The bedrock fissure aquifer located in southern, eastern and northern parts of the study area have a shallow water levels, which mainly recharge river water and Quaternary pore aquifer.

### Conditions of seepage water in the mine area

The Dagushan iron mine is a huge open-pit mine with an excavation area of approximately 2 square kilometres at present (Fig. 2b). In addition, an underground roadway with a length of 1314 m was excavated in the northwestern part of the mine (Fig. 2c) to transport ore. The long-term mining activities have seriously affected the groundwater seepage field and the hydrogeochemical regime in the mining area, which has led to groundwater seeping into the pit, resulting in multiple groundwater seepages in the mining area. As shown in Fig. 2c, there are two water seepage points along the rock fracture in the 2# roadway (J1、J2), which are located at 330 m and 580 m away from the entrance of mine roadway. The 3# roadway has a large seepage area between 1080 m and 1150 m away from the entrance of mine roadway. There are many water seepage points, and the water seepage flow rate is large than that of the 2# roadway.

There are three seepage points on the slopes of the open pit (Fig. 2b). Two of them (K1, K2) are on the south side of the pit, another one is located on the west side of the pit (K3). It can be seen from Fig. 2b that spatial position of K3 is close to the 3# roadway. To control the water seepage problem and supply the mining production water, there is a pumping well on the southeastern side of the mine pit to pump groundwater all year-round. However, the huge depression funnel formed by long-term open-pit excavation has led to the continuous collection of surrounding groundwater towards the direction of the mine, which not only brings danger to mining activities but also causes ecological damage such as declining groundwater levels and cutting off of the river around the mining area.

## Materials and methods

### Sampling and analysis

In this study, 33 groundwater samples, 5 river water samples and 8 mine seepage water samples (Fig. 2a) were collected in April 2019. The groundwater samples were collected from wells for domestic and agricultural purposes at different depths, including 8 alluvial pore water samples, 8 eluvium pore water samples, and 17 bedrock fissure water samples. The mine seepage water samples were collected from 3 seepage points in the pit, 2 seepage points in the 2# roadway and 3 seepage points in the 3# roadway. The samples were stored in 250 ml polyethylene bottles and refrigerated until chemical analysis. The total dissolved solids (TDS) and pH were measured in-situ using a portable multiparameter water quality meter (HANNA, HI9828). The samples were analyzed at the laboratory of the Liaoning Metallurgical Geological Exploration Research Institute. Major anions (SO_4_^2^ˉ, Clˉ, and NO_3_ˉ) were measured with an ion chromatograph (DX-120, Dionex). The alkalinity as HCO_3_ˉ was measured using the titrimetric method. Concentrations of major cations (Na^+^, K^+^, Ca^2+^, and Mg^2+^) were measured by inductively coupled plasma mass spectrometry (ICP-MS). The percentage of charge balance error (%CBE) calculated showed a result of less than 5%, which means that the accuracy of the measurement meets the quality requirements. The number of sampling points and the analysis results are shown in Table 1. Then, a Piper diagram was drawn with Aquachem (version 1.1) to classify the hydrochemical types of the samples.

**Table 1:** The analysis results for water samples in the study area (all hydrochemical compositions are in units of mg/L except for pH).

No. of samples	Sample type ID	pH	Ca^2+^	Mg^2+^	Na^+^ + K^+^	HCO_3_ˉ	SO_4_^2^ˉ	Clˉ	NO_3_ˉ	TDS
D01	2	6.71	42.26	14.80	14.99	117.20	96.15	34.55	7.00	267.99
D02	2	7.50	54.71	45.26	21.08	175.63	178.91	34.59	5.00	427.51
D03	3	7.16	39.79	33.19	17.34	116.99	95.43	34.12	44.00	322.94
D04	3	7.29	54.31	24.62	15.03	161.37	107.35	25.87	7.00	315.45
D05	2	8.03	51.29	23.87	23.53	117.12	167.00	34.49	18.00	378.06
D06	2	7.61	72.41	34.82	16.46	190.45	191.23	34.23	38.00	482.46
D07	3	6.98	47.99	25.02	15.21	205.10	71.61	34.49	18.00	315.99
D08	3	7.95	51.12	34.28	26.98	233.98	119.30	25.78	19.00	394.99
D09	2	7.27	53.70	32.83	20.89	175.37	104.94	47.23	36.00	384.01
D10	4	7.18	52.79	37.30	22.25	204.96	106.83	42.97	25.00	390.77
D11	2	7.07	73.92	62.25	27.83	293.17	191.12	50.93	11.00	564.51
D12	3	7.23	67.15	49.78	30.14	293.05	106.94	60.37	6.00	467.61
D13	2	7.43	60.03	50.98	36.20	234.40	119.31	64.37	44.00	493.39
D14	1	7.03	53.02	42.28	34.24	146.31	143.14	51.53	70.00	467.69
D15	2	6.81	53.88	48.97	26.20	87.90	202.74	51.61	73.00	501.83
D16	3	8.05	48.27	14.92	13.38	160.83	83.52	30.18	11.00	282.28
D17	3	7.14	78.45	53.63	22.48	146.37	214.78	82.12	78.00	602.60
D18	4	7.85	129.74	119.37	103.74	366.25	691.48	34.48	11.00	1,274.55
D19	3	7.18	78.98	27.15	22.69	175.60	107.12	44.12	60.00	428.11
D20	3	7.50	114.40	75.43	29.73	234.40	370.05	65.01	0.60	772.00
D21	3	7.07	74.61	57.33	19.67	322.47	131.22	51.75	24.00	519.93
D22	3	7.51	66.37	29.84	12.54	234.26	155.06	17.25	10.00	408.45
D23	3	7.68	66.38	59.68	18.03	176.15	166.95	42.83	108.00	551.36
D24	3	7.54	81.46	24.87	15.55	190.32	214.65	34.35	24.00	490.52
D25	1	7.07	90.51	70.03	39.25	205.02	274.25	103.05	47.00	578.53
D26	1	7.62	79.58	54.34	42.12	263.70	190.67	68.99	11.00	579.33
D27	4	7.28	60.34	59.68	81.18	276.77	155.04	86.25	15.00	601.36
D28	1	6.89	82.20	49.32	30.49	131.85	274.25	34.43	131.00	668.11
D29	1	7.29	129.32	85.03	80.13	249.05	321.95	133.68	146.00	1,020.28
D30	3	7.08	48.27	24.87	16.99	117.20	119.31	34.50	47.00	349.73
D31	3	7.14	52.98	49.70	12.60	146.50	101.44	21.56	8.00	319.63
D32	1	7.14	108.62	106.23	46.08	307.65	405.42	64.93	73.00	961.27
D33	4	7.80	116.88	98.05	107.09	410.21	262.34	238.33	0.40	1,028.69
D34	1	7.79	171.60	92.02	130.07	454.15	405.21	176.81	9.00	1,213.79
D35	4	7.73	119.17	117.18	97.98	380.90	464.98	120.75	14.00	1,124.81
D36	3	7.07	69.39	54.71	24.56	204.87	238.52	38.47	3.00	531.75
D37	3	6.77	65.85	49.88	20.79	161.05	214.63	47.44	41.00	520.70
D38	1	6.38	67.88	57.30	52.57	160.97	214.55	81.94	12.00	547.05
J1	5	8.19	84.48	49.74	15.88	234.13	226.58	52.03	9.00	554.75
J2	5	8.18	89.53	60.34	19.33	263.70	179.15	77.62	20.00	577.70
J3	5	7.98	131.81	97.83	61.83	351.60	322.34	137.99	87.00	1,014.55
J4	5	7.96	139.27	66.12	67.57	322.30	309.85	120.75	21.00	886.28
J5	5	8.13	134.29	69.39	57.06	292.36	321.77	103.49	38.00	924.91
K1	6	8.17	109.42	76.02	55.21	219.75	417.28	29.99	89.00	887.62
K2	6	8.16	159.16	89.63	56.10	119.16	631.88	29.83	30.00	1,058.21
K3	6	7.96	144.24	82.67	66.19	351.60	369.64	119.36	1.00	960.45

Sample type ID: 1-Alluvial pore aquifer, 2-Eluvium pore aquifer, 3-Bedrock fissure aquifer, 4-River, 5-Water seepage points in the roadway, 6-Water seepage points in the pit.

### Multivariate statistical analysis

The multivariate statistical analysis methods (i.e., HCA and FDA) were employed to investigate the acquired data and identify mine water sources. The hydrochemical similarity of groundwater between different aquifers was qualitatively analyzed by Q-mode HCA, and the seepage water sources were quantitatively identified by the discriminant model established by FDA.

Q-mode HCA classifies samples into typical hydrochemical groups or subgroups in which the group members are similar to each other, but groups are distinct from each other. Using the Ward’s linkage to classify the samples and taking Euclidean square distance as a measure of similarity is a common method for classification of hydrochemical samples in different aquifers^34,45^. The basic outputs of this method is a dendrogram, explaining the main processes of groundwater evolution and evaluate the hydraulic connectivity between aquifers by combining the grouping of samples in the dendrogram and their spatial distribution positions.

FDA is an important method for the reduction and classification of hydrochemical data dimensionality. Taking the predicted samples as training samples, FDA distinguishes unknown samples by establishing discriminant functions according to the principle of maximum distance between groups and minimum distance within groups^46^. In the process of discriminant analysis, the prediction variables are selected in order to establish the discriminant function more accurately. Therefore, it is necessary to conduct statistical tests on the raw data to ensure that the variables in the discriminant equation are significantly different^35^. Then, the discriminant functions are established by linear combination of the prediction variables from the measured samples. Finally, the distance between the unknown samples and each training sample centre is calculated according to the discriminant function to determine the class of the unknown samples. At the same time, the discriminant function also reclassifies the training samples to strengthen the understanding of the recharge relationship between different aquifers. In this study, SPSS Statistics 23 software was used to perform HCA and FDA calculations.

## Results and discussion

### Hydrochemical characteristics

#### Hydrochemical parameter statistics

**Figure 3:**
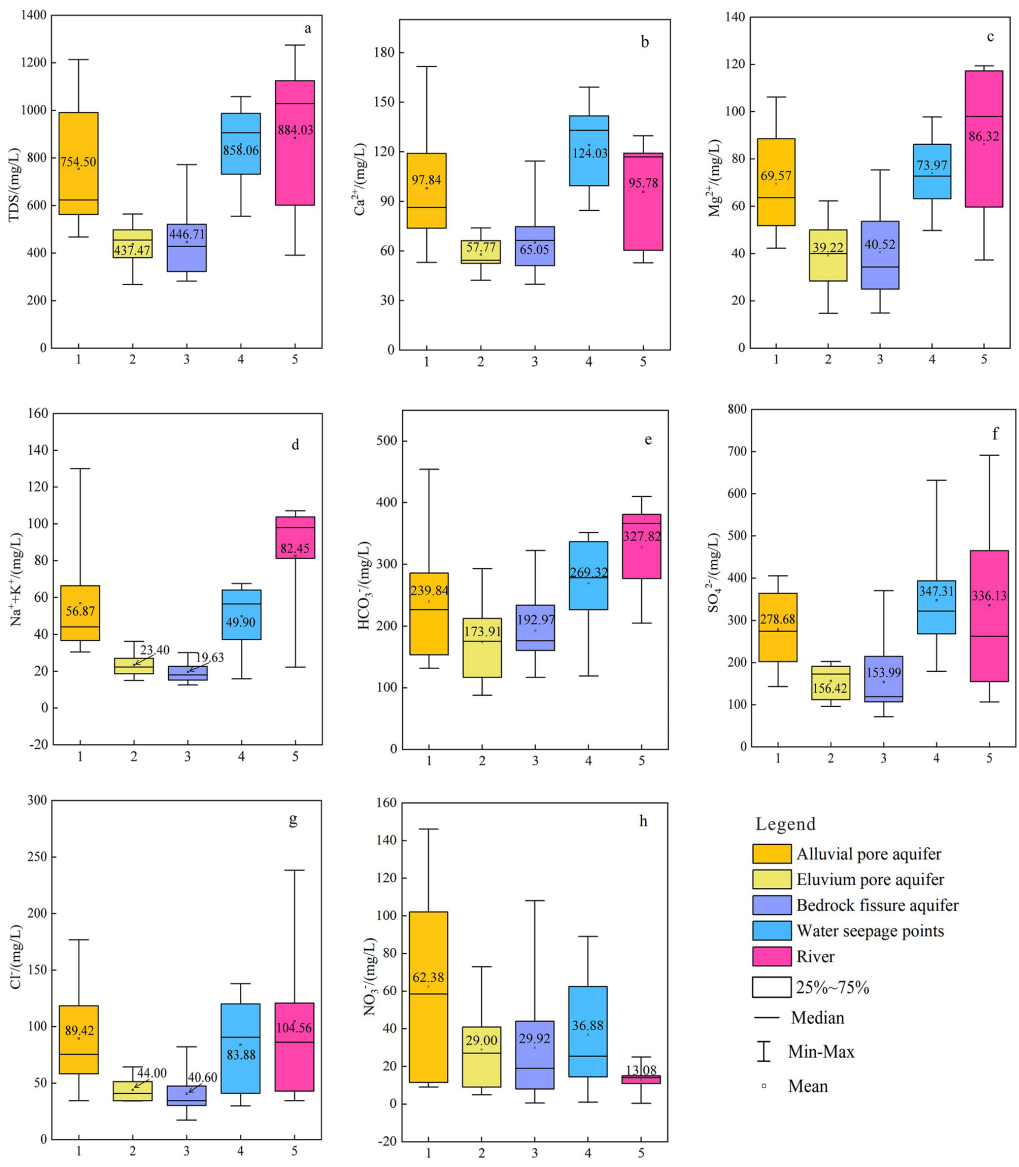
Boxplots of the water samples **(a–h)**.

According to Table 1, most of the samples in the study area are weakly alkaline, with an average pH of 7.45. Boxplots (Fig. 3a–h) are used to analyze the variation of the concentrations of main ion and TDS in groundwater, river, and seepage waters in the study area. From top to bottom, the boxplots show the maximum value, upper quartile, average or median, lower quartile, and minimum value for these concentrations. Since K^+^ shows very low concentrations and similar chemical characteristics to Na^+^, the Na^+^ and K^+^ are combined in the analysis of hydrochemical characteristics and water source identification^47,48^.

It can be seen from the boxplots that the concentrations of main ion and TDS from the alluvial pore aquifer are highly variable compared with those of the eluvium pore aquifer and bedrock fissure aquifer, which shows that the alluvial pore water is greatly affected by the external environment. The range of variability for the major ion concentrations of samples from water seepage points is large, indicating that the source of seepage water is relatively complicated. By comparing the average concentrations of ions in different water types, it can be seen that the cation mass concentrations in most samples occur in the order of Ca^2+^  > Mg^2+^  > Na^+^  + K^+^. However, the concentrations of anions differ; in alluvial pore water, river water and seepage water these concentrations occur in the order of SO_4_^2^ˉ > HCO_3_ˉ > Clˉ > NO_3_ˉ, while in bedrock fissure water and eluvium pore water they occur in the order of HCO_3_ˉ > SO_4_^2^ˉ > Clˉ > NO_3_ˉ.

According to hydrochemical parameter statistics, the hydrochemical characteristics of bedrock fissure water and eluvium pore water are similar, and those of alluvial pore water and river water are similar. The concentrations of ions and TDS in seepage water falls between the values of groundwater and surface water values in the study area, so it can be speculated that seepage water is produced by the mixture of different water bodies, and the source of seepage water is relatively complicated.

#### Hydrochemical types

**Figure 4:**
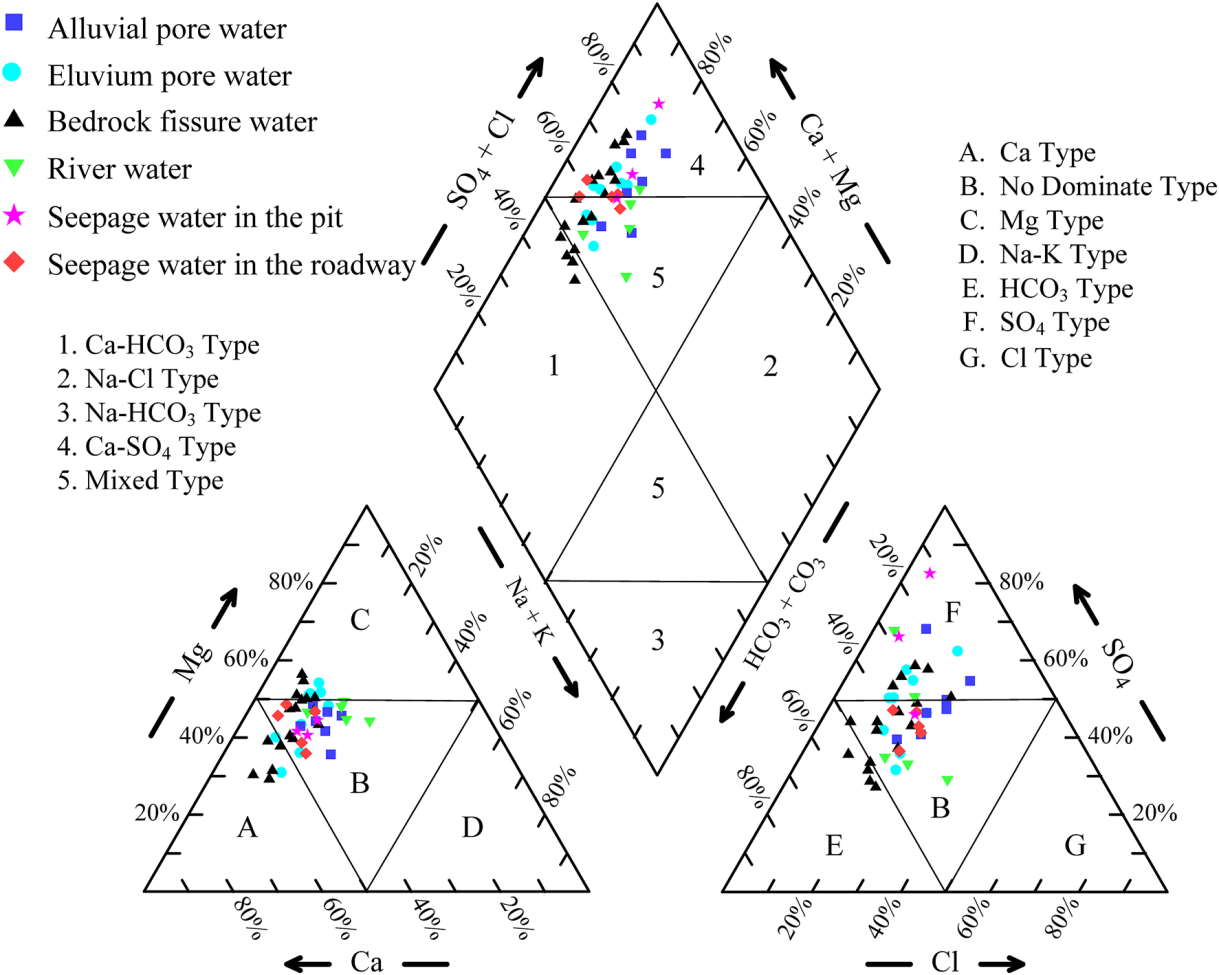
Piper diagrams of water samples in the study area.

The evolution of hydrochemical types in different aquifers indicates the potential flow paths and hydraulic connections between different aquifers, which is of great significance for identifying the sources of mine water recharge^49^. The Piper diagram in this study describes the overall hydrogeochemical types of water samples. As shown in Fig. 4, only a number of samples taken from the bedrock fissure aquifer located in zone 1, the water samples from the study area are primarily located in zones 4 and 5, suggesting that the hydrochemical types are mainly HCO_3_·SO_4_-Ca·Mg, SO_4_·HCO_3_-Ca·Mg and SO_4_-Ca·Mg. The samples from the bedrock fissure aquifer are similar to those of the eluvium pore aquifer (Fig. 4), and can be seen from the boxplots (Fig. 3) that the concentrations of major ions in the bedrock fractured aquifer and the eluvium pore aquifer samples are similar, which indicates that they have possible hydraulic connection or similar source of water body.

It is worth noting that the samples (J3, J4, and J5) from the 3# roadway water seepage points show relatively consistent characteristics. Their distribution positions in the Piper diagram (Fig. 4) are close to some river water and alluvial pore water samples, which indicates that the seepage water of the 3# roadway may have a recharge relationship with shallow alluvial pore water and surface water. However, the samples from the 2# roadway water seepage points are characterized by low concentrations of Na^+^  + K^+^ and their distribution positions are close to some bedrock fissure water samples in Fig. 4, indicating that the 2# roadway seepage water may be supplied by bedrock fissure water. The seepage water samples from the pit are scattered in the Piper diagram. The samples from the southern side of the pit (K1 and K2) show a milliequivalent percentage of SO_4_^2^ˉ of more than 60%, and their hydrochemical type is SO_4_-Ca·Mg. However, the samples from the western side of the pit and the 3# roadway seepage water samples overlap in Fig. 4, which shows that they may supply from a similar source.

### Hierarchical cluster analysis (HCA)

**Figure 5:**
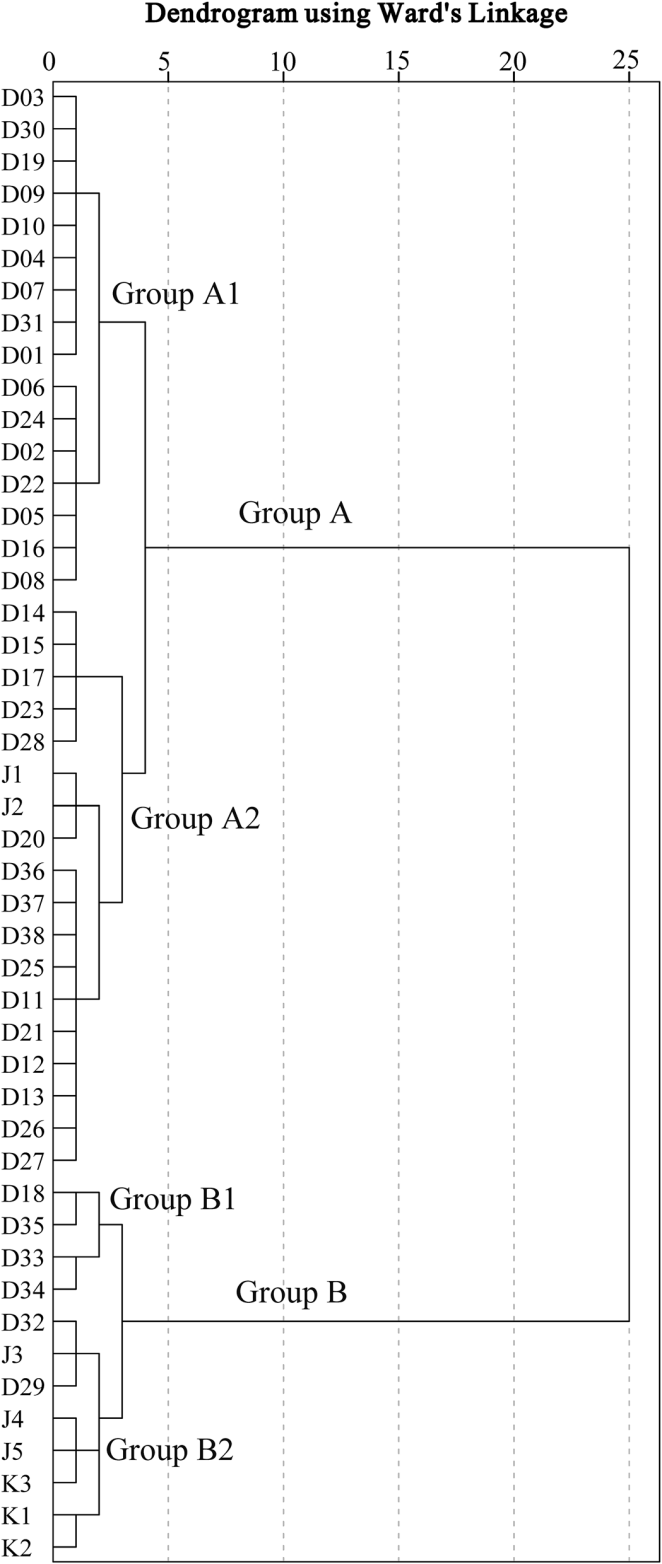
Dendrogram of Q-mode HCA.

**Figure 6:**
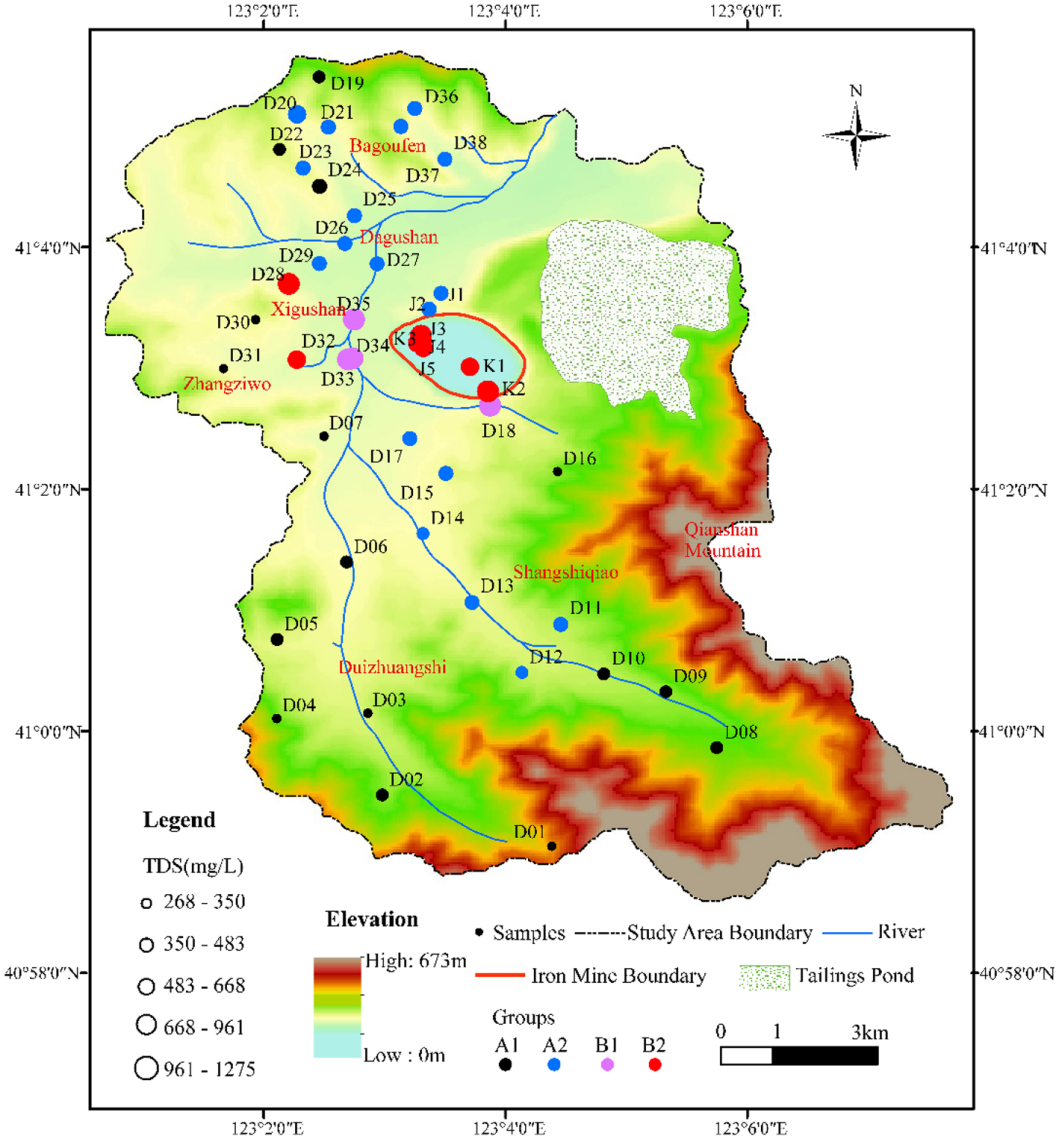
Spatial distributions of the water groups defined by HCA and the concentrations of TDS in the water samples. The diagram was generated using ArcGIS 10.2 (https://www.esri.com).

**Figure 7:**
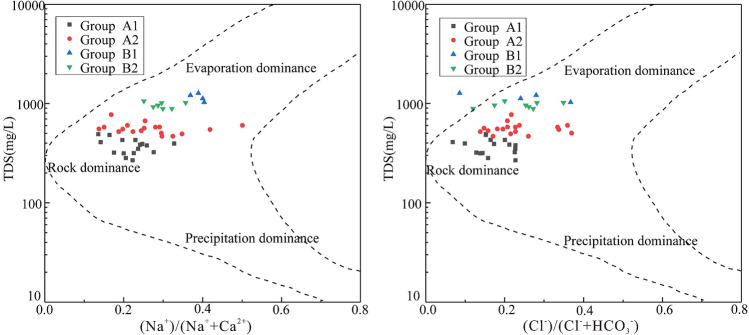
Gibbs diagrams of the water groups defined by HCA.

In this study, HCA was applied by using Ward’s linkage to classify the samples and taking Euclidean square distances for similarity measurements. The hydrochemical data (Ca^2+^, Mg^2+^, Na^+^  + K^+^, HCO_3_ˉ, SO_4_^2^ˉ, Clˉ, NO_3_ˉ, pH, and TDS) from each water sample were standardized as input variables in the analysis. The HCA results are presented as a dendrogram in Fig. 5. According to the characteristics of the dendrogram, the samples from the study area were classified into two major groups (A and B). Additionally, the groups A and B were further composed of two subgroups (A1 and A2, B1 and B2). The spatial distributions of the groups defined by HCA and the concentrations of TDS in the water samples are shown in Fig. 6. To illustrate hydrochemical differences between the groups and to determine the cause of seepage water in the mine, the cluster groups are plotted into a Gibbs diagram (Fig. 7).

Group A1 contains a total of 16 samples (10 bedrock fissure water samples, 5 eluvium pore water samples and 1 river water sample) mainly distributed in the hilly and mountainous areas with higher altitudes in the study area (Fig. 6). The hydrochemical type is mainly HCO_3_·SO_4_-Ca·Mg, which is primarily affected by water–rock interaction (Fig. 7). The average content of TDS in this group is 372.43 mg/L, and the salinity is low, indicating that the residence time of groundwater in the aquifer is short, which represent the characteristics of groundwater recharge area.

Group A2 contains 39.13% of all samples, including most samples from the eluvium pore aquifer and some from the bedrock fissure aquifer. In addition, it also contains the 2# roadway seepage water samples (J1 and J2). The samples in this group are mainly distributed in the transition zone from the mountainous area to the plain (Fig. 6). The contents of TDS varies from 467.61 mg/L to 772.00 mg/L, and the hydrochemical type is mainly SO_4_·HCO_3_-Ca·Mg. Compared with group A1, the content of TDS for the samples in this group is higher and they are subjected to some degree of evaporation (Fig. 7), which represents the characteristics of run-off area.

Group B contains a relatively small number of samples, mainly from seepage water in the mining area, as well as the alluvial pore water and river water samples from the western side of the study area. Group B1 includes three river water samples (D18, D33, and D35) and one alluvial pore water sample (D34) with the contents of TDS greater than 1 g/L. It is speculated from Fig. 7 that the high salinity may be due to some degree of evaporation. Similar hydrochemical characteristics indicate that there is a recharge relationship between river water and shallow alluvial pore water in this area.

Group B2 contains the seepage water samples in the 3# roadway (J3, J4, and J5) and the western side of the pit (K3), as well as alluvial pore water samples (D29, D32) which are located on the western side of the study area. It can be seen from Fig. 7 that the samples from this group are mainly controlled by water–rock interaction and evaporation, and the contents of TDS are similar (Table 1). It can be seen from the above analysis that there is a strong hydraulic connection between the seepage water in the 3# roadway and the alluvial pore water.

In addition, although the seepage water samples on the southern side of the pit (K1, K2) also belong to group B2, it can be inferred that their source is different from the western side of the mine according to their distribution location (Fig. 1b). The hydrochemical type of K1 and K2 is SO_4_-Ca·Mg, is consistent with groundwater samples (D15 and D17) on the southern side of the pit. However, the ion concentrations of K1 and K2 are significantly higher than those of groundwater samples, which were similar to a river sample (D18) from the southern side of the pit (Table 1). In particular, the concentrations of SO_4_^2^ˉ in K1, K2 and D18 were abnormally high (Fig. 3f), reaching 417.28 mg/L, 631.88 mg/L and 691.48 mg/L, respectively. This shows that the seepage water of the southern pit is caused by the leakage of river water and groundwater along the water-conducting fissure in the southern side of the mine due to the excavation of the mine.

### Fisher’s discriminant analysis (FDA)

For this analysis, the predicted water samples are divided into four groups: (1) Alluvial pore water, (2) Eluvium pore water, (3) Bedrock fissure water, and (4) River water. In addition, the samples from seepage water points are currently considered to be the uncertain group. To ensure the accuracy of the results in the process of discriminant analysis, the predicted sample variables must have significant differences. Therefore, before performing the discriminant analysis, it is necessary to select the variables according to whether there are differences among the group means. The original indicators Ca^2+^, Mg^2+^, Na^+^  + K^+^, HCO_3_ˉ, SO_4_^2^ˉ, Clˉ, NO_3_ˉ, and pH of the water samples were selected for the average test and analysis. The results are shown in Table 2, which smaller Lambda values indicate the greater the contributions to the discriminant function^35^.The last column gives the results of the null hypothesis test of equality of means in each group. No significant differences in NO_3_ˉ and pH among groups are seen, so they are excluded from the discriminant analysis variables.

**Table 2:** Equivalence test of group means.

Original indicators	Wilks’ Lambda	Significance
Ca^2+^	0.67	0.00
Mg^2+^	0.56	0.00
HCO_3_ˉ	0.68	0.00
SO_4_^2^ˉ	0.68	0.00
Clˉ	0.65	0.00
NO_3_ˉ	0.81	**0.06**
Na^+^ + K^+^	0.40	0.00
pH	0.90	**0.32**

Significant values are in bold.

According to the selected prediction variables (Table 2), the discriminant functions (Eq. 1) for seepage water are obtained by Fisher’s discriminant analysis method^50^, given as follows:

1 $$ \left\{ \begin{gathered} F1 = - 0.071{\text{ Ca}}^{2 + } + 0.003{\text{ Mg}}^{2 + } + 0.004{\text{ HCO}}_{3}^{ - } + 0.009{\text{ SO}}_{4}^{2 - } + \, 0.012{\text{ Cl}}^{ - } + 0.055 \, \left( {{\text{Na}}^{ + } + {\text{K}}^{ + } } \right) - 0.280 \hfill \\ F2 = - 0.101{\text{ Ca}}^{2 + } - 0.050{\text{ Mg}}^{2 + } + 0.020{\text{ HCO}}_{3}^{ - } + 0.018{\text{ SO}}_{4}^{2 - } + \, 0.022{\text{ Cl}}^{ - } - 0.026 \, \left( {{\text{Na}}^{ + } + {\text{K}}^{ + } } \right) \, + 1.581 \hfill \\ F3 = 0.032{\text{ Ca}}^{2 + } + 0.024{\text{ Mg}}^{2 + } + 0.012{\text{ HCO}}_{3}^{ - } - 0.009{\text{ SO}}_{4}^{2 - } - \, 0.022{\text{ Cl}}^{ - } - 0.010 \, \left( {{\text{Na}}^{ + } + {\text{K}}^{ + } } \right) - 2.663 \hfill \\ \end{gathered} \right. $$

The percentage of cumulative variance of discriminant functions F1 and F2 is 97.6%, which means that most of the sample information can be explained using F1 and F2.

**Figure 8:**
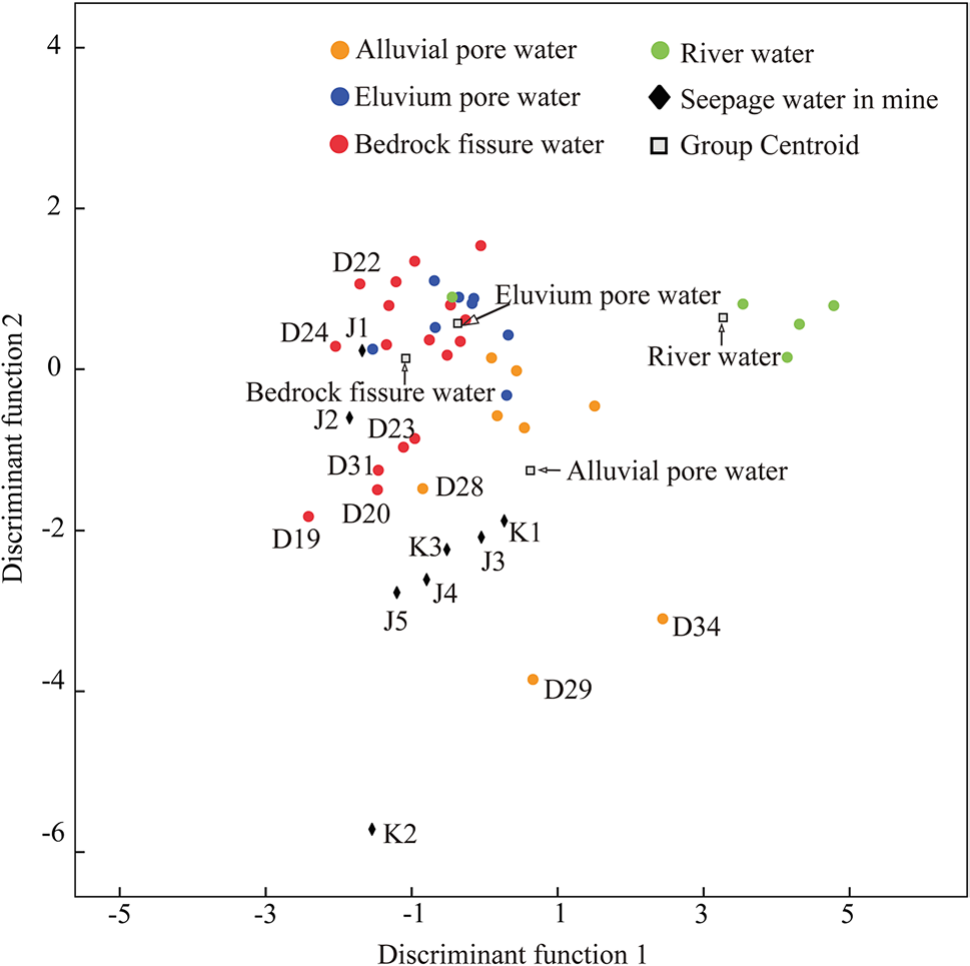
Scatter plot of each sample score with the first and second discriminant functions.

Table 3 lists the misjudgment analysis results for 38 training samples and the results of seepage water source identification by discriminant functions. From Table 3, two samples (D06, D11) from eluvium pore aquifer were misjudged to be from bedrock fissure aquifer, and six samples (D03, D08, D16, D30, D36 and D37) from the bedrock the fissure aquifer were misjudged to be from the eluvium pore aquifer. In addition, in the scatter plot (Fig. 8) calculated by the discriminant functions F1 and F2, the samples from the eluvium pore aquifer and the bedrock fissure aquifer basically overlap. This indicates that there is a possible hydraulic connection or similar source of water body between the eluvium pore aquifer and the bedrock fissure aquifer, which is consistent with the previous hydrochemical characteristics analysis.

**Table 3:** Misjudgement analysis of training samples and seepage water source identification results.

Misjudged water sample	Original group	Predicting group	Samples of seepage water	Discriminant results
D14	1	2	K1	1
D26	1	2	K2	1
D06	2	3	K3	1
D11	2	3	J1	3
D03	3	2	J2	3
D08	3	2	J3	1
D16	3	2	J4	1
D30	3	2	J5	1
D36	3	2		
D37	3	2		
D10	4	2

For the seepage water in the mining area, while the 2# roadway seepage water (J1, J2) was judged to be from the bedrock fissure aquifer, the other seepage water was identified as originating from the alluvial pore aquifer (Table 3). In Fig. 8, the distance between the location of the 2# roadway seepage water samples and the centre of the bedrock fissure water samples is shorter than the distance to the central locations of other water sample groups. The locations of J1 and J2 are close to those of the D20, D22, D23, and D24 samples from the bedrock fracture aquifer in the northern part of the study area (Fig. 8), which is consistent with the results of HCA analysis. This indicates that there is a hydraulic connection between the 2# roadway seepage water and the bedrock fracture aquifer. The study of hydrochemical characteristics also shows that the seepage water of the 2# roadway is similar to the bedrock fissure water, with an average contents of TDS of 554.75 mg/L and 577.70 mg/L for J1 and J2, respectively. The field investigation results show that the seepage flow rate is small. The above analysis shows that J1 and J2 are caused by underground roadway excavation exposing the bedrock fissure aquifer, and groundwater is discharged through water-conducting fissures, resulting in water seepage.

Compared with the seepage water of the 2# roadway, the 3# roadway seepage water (J3, J4, and J5) and pit seepage water (K3) in the deeper underground position are also located in the bedrock fissure aquifer. However, differing from the seepage water of 2# roadway, the seepage water samples of the 3# roadway are characterized by a large seepage flow rate and high salinity. In Fig. 8, they are distributed between the alluvial pore water and bedrock fissure water in the western part of the mine. Combined with the HCA results and the geological structure of the study area, it is inferred that the faults F14 and F15 connect the shallow alluvial pore aquifer and the bedrock fissure aquifer, and the concentrated seepage waters occur in the excavated roadway face and pit. In addition, as previously discussed, the river water has a hydraulic connection with the shallow alluvial pore water in the western part of the mining area. Therefore, a part of the serious water seepage in the 3# roadway might be caused by the river supplying shallow alluvial pore water, and then leading to serious water seepage in the mining area through the seepage channel. The field investigation results show that the river flow near the pit is obviously smaller than that at other locations, which is another can evidence for this conclusion.

## Conclusions

In this paper, the sources of seepage water in an open-pit mine are studied by combining the analysis of hydrochemical characteristics and multivariate statistics. The following conclusions have been reached:

(1) The hydrochemical characteristics of groundwater in the bedrock fissure aquifer and the eluvium pore aquifer are similar, and there is a possible hydraulic connection or similar source of water body between them. Shallow alluvial pore water and river water are affected by evaporation to a certain extent, resulting in higher salinity. There are large differences in the hydrochemical characteristics of seepage water at different locations in mining areas, indicating that the sources of the mining area water are complex.

(2) Water seepage in the 2# roadway is due to the excavation penetrating the water-conducting fissures in the bedrock fissure aquifer, and groundwater recharge in the northern part of the mine causing seepage. The fault connects the shallow alluvial pore aquifer and bedrock fissure aquifer, causing considerable water seepage in the 3# roadway and the western side of the pit. The source of water on the southern side pit comes from the river and groundwater on the southern side of the mine.

(3) Hydrogeochemical discrimination by combining groundwater hydrochemical characteristics, HCA, and FDA can improve the discrimination accuracy of complex seepage water sources of the open-pit mine, which can improve the pertinence and effectiveness of efforts to prevent problems associated with mine water seepage.
